# Validity and utility of blood tumor mutational burden (bTMB) is dependent on circulating tumor DNA (ctDNA) shed: SCRUM-Japan MONSTAR-SCREEN

**DOI:** 10.1016/j.jlb.2023.100003

**Published:** 2023-08-10

**Authors:** Saori Mishima, Yoshiaki Nakamura, Hanna Tukachinsky, Hiroya Taniguchi, Shigenori Kadowaki, Ken Kato, Eiji Oki, Taroh Satoh, Daisuke Aoki, Kentaro Yamazaki, Taito Esaki, Makoto Ueno, Tomohiro Nishina, Yu Sunakawa, Tadamichi Denda, Hideaki Bando, Naomi Kuramoto, Satoshi Horasawa, Hikaru Abutani, Jessica K. Lee, Russell W. Madison, Geoffrey R. Oxnard, Takayuki Yoshino

**Affiliations:** a Department of Gastroenterology and Gastrointestinal Oncology, National Cancer Center Hospital East, Kashiwa, Japan; b Translational Research Support Section, National Cancer Center Hospital East, Kashiwa, Japan; c Foundation Medicine, Massachusetts, USA; d Department of Clinical Oncology, Aichi Cancer Center, Aichi, Japan; e Department of Head and Neck, Esophageal Medical Oncology, National Cancer Center Hospital, Tokyo, Japan; f Department of Surgery and Science, Graduate School of Medical Sciences, Kyushu University, Fukuoka, Japan; g Department of Frontier Science for Cancer and Chemotherapy, Graduate School of Medicine, Osaka University, Osaka, Japan; h Department of Obstetrics and Gynecology, Keio University School of Medicine, Tokyo, Japan; i Division of Gastrointestinal Oncology, Shizuoka Cancer Center, Shizuoka, Japan; j Department of Gastrointestinal and Medical Oncology, National Hospital Organization Kyushu Cancer Center, Fukuoka, Japan; k Department of Gastroenterology, Hepatobiliary and Pancreatic Medical Oncology Division, Kanagawa Cancer Center, Kanagawa, Japan; l Department of Gastrointestinal Medical Oncology, Shikoku Cancer Center, Ehime, Japan; m Department of Clinical Oncology, St. Marianna University School of Medicine, Kanagawa, Japan; n Division of Gastroenterology, Chiba Cancer Center, Chiba, Japan; o Chugai Pharmaceutical Co., Ltd, Tokyo, Japan

**Keywords:** Tumor mutational burden, Blood tumor mutational burden, Liquid biopsy, Circulating tumor DNA, Precision oncology, Immunotherapy

## Abstract

**Background:**

The tumor mutational burden (TMB) is a genomic biomarker associated with the benefits from immune checkpoint inhibitors (ICIs) cancer therapy. An elevated blood TMB (bTMB) in circulating tumor DNA (ctDNA) represents a compelling non-invasive diagnostic approach; however, the validity and utility of this emerging biomarker across cancer types have not been examined.

**Patient and methods:**

The blood and tissue TMB was measured in a large pan-tumor clinical cohort and the MONSTAR-SCREEN observational study (UMIN000036749) using the FoundationOne Liquid CDx and FoundationOne CDx assays. A subset of the MONSTAR-SCREEN cohort was used to evaluate the association between the bTMB and the efficacy of ICIs therapy.

**Results:**

The majority of cancer types showed similar prevalence of TMB≥10 mutations/megabase and bTMB≥10. There was high concordance between bTMB and TMB in blood and tissue biopsy from the same patient when the plasma tumor fraction (TF) was at least 1% (Spearman's coefficient 0.74 and > 80% sensitivity to detect TMB-high). High microsatellite instability (MSI-H) was detected by ctDNA with 79% sensitivity when TF was at least 1%, but only 6% sensitivity when TF was <1%. Among patients with bTMB≥14 and elevated TF≥10% treated with ICIs, there was a trend toward a longer progression-free survival and overall survival compared with patients with bTMB<14 and elevated TF≥10% (HR, 0.62 [95%CI, 0.39–0.98]; p = 0.04 and HR, 0.60 [95%CI, 0.34-1.1]; p = 0.05).

**Conclusions:**

Our findings suggest that an elevated bTMB is correlated with elevated TMB and represents a pragmatic biomarker for assessing ICIs benefits. The utility of this biomarker is likely to be associated with high TF levels, informing future prospective investigations.

## Introduction

Immune checkpoint inhibitors (ICIs) significantly prolong patient survival in many types of cancers [1]. Extensive investigation has resulted in a range of potential biomarkers. A prospective tumor-agnostic study, KEYNOTE-158, which was a single-arm, phase II multi-cohort study of pembrolizumab for previously treated advanced solid tumors showed the association of tumor mutational burden (TMB) with the objective response rate (ORR) of pembrolizumab. Based on the results of this trial [2], the U.S. Food and Drug Administration (FDA) approved pembrolizumab as a therapy for all solid tumors with tissue TMB equal to or greater than 10 mutations/megabase (mut/Mb), as measured by the FoundationOne® CDx assay (F1CDx). Real-world data from over 8000 patients with 24 cancer types also indicated a more favorable overall survival on ICI in patients with high TMB tumors determined by F1CDx than low TMB, across the heterogeneous cohort as well as individual cancer types like breast, colorectal, endometrial, gastric, head & neck, melanoma, non-small cell (NSCLC) and small cell lung, and urothelial cancer [3].

Liquid biopsy technologies for the analysis of circulating tumor DNA (ctDNA) have evolved rapidly in recent years with the regulatory approval of multigene next-generation sequencing (NGS) assays for profiling advanced solid tumors [4]. It has been suggested that liquid biopsy may resolve issues associated with conventional tumor tissue biopsy, as liquid biopsy allows genomic profiling without tumor tissue samples and also as the turnaround time to return test results is shorter [5]. Moreover, liquid biopsies may be less susceptible to sampling biases associated with single-site tissue biopsies [6,7]. High blood TMB (bTMB) can be calculated from ctDNA samples, and elevated bTMB, assessed using different CGP assays, has been reported to be associated with benefits from ICIs in patients with advanced NSCLC [[8], [9], [10], [11]].

However, the clinical performance of bTMB measurement has not yet been fully described, nor has the utility of this biomarker for guiding therapy across solid tumors. In Japan, a MONSTAR-SCREEN study is being conducted to investigate these questions. MONSTAR-SCREEN has been a prospective monitoring study of cancer-related genomic alterations and the gut microbiome in advanced solid tumors using ctDNA sequencing since July 2019 (UMIN 000036749). By studying a large routine clinical care cohort and the MONSTAR-SCREEN cohort, we aimed to (i) assess the landscape of bTMB in various cancer types, (ii) evaluate the concordance of tissue and blood mutational burden, and (iii) assess the association of bTMB with ICIs therapy outcomes.

## Methods

### Routine clinical care cohort

CGP results reported during routine clinical care were used in this study. Approval for this study, including a waiver of informed consent and Health Insurance Portability and Accountability Act waiver of authorization, was obtained from the Western Institutional Review Board (Protocol 20152817). No clinical characteristics or information on therapy use were available for this cohort.

**Fig. 1:**
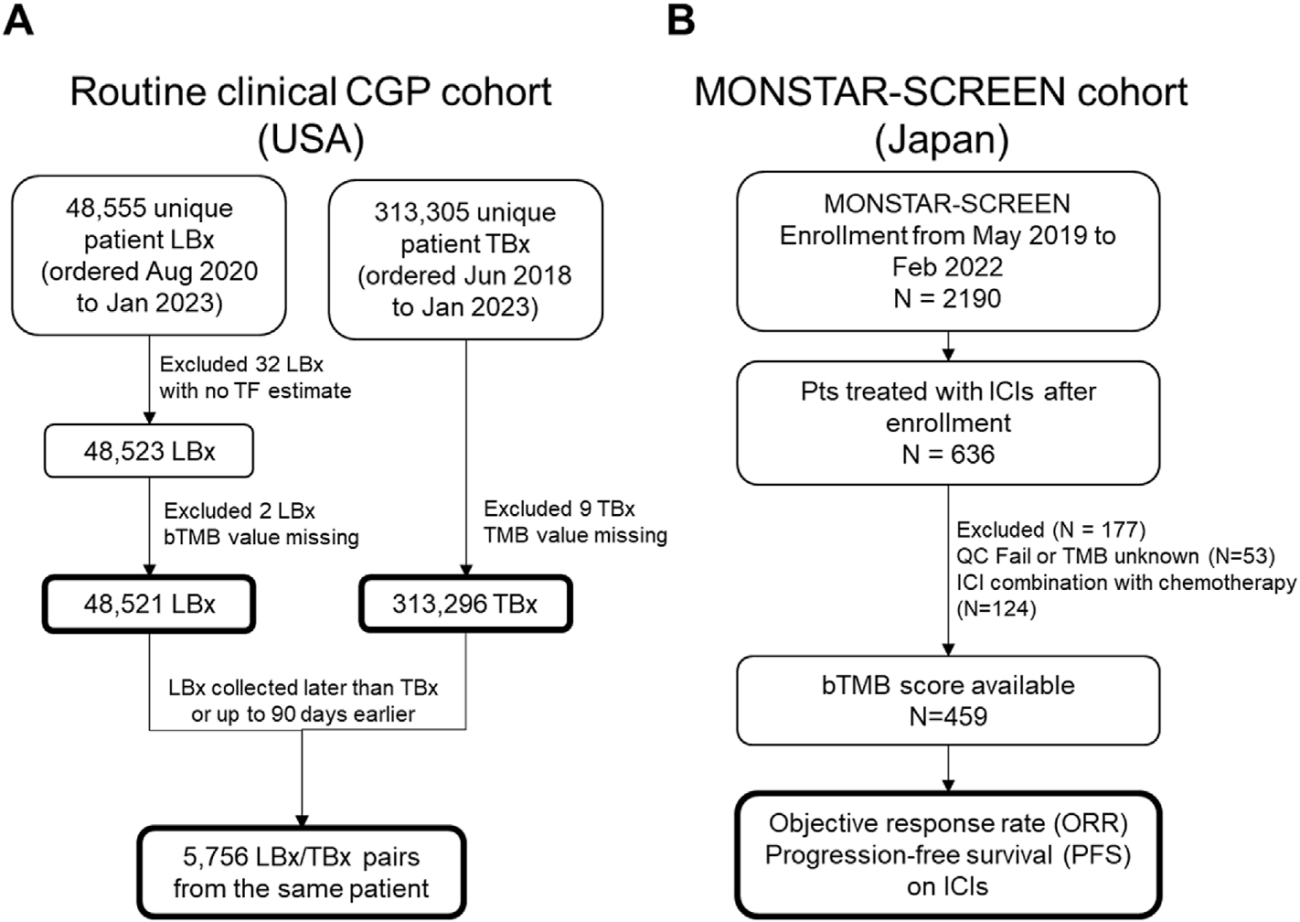
CONSORT diagrams of a retrospectively analyzed clinical cohort of CGP performed during routine clinical care in the USA (A) and the prospectively analyzed MONSTAR-SCREEN cohort from Japan (B). LBx: Liquid biopsy. TBx: Tissue biopsy. QC: quality control.

Liquid biopsies were collected from 48,521 unique patients (August 2020–Jan 2023) and profiled using FoundationOne® Liquid CDx (F1LCDx). Tissue biopsies from 313,296 unique patients were profiled using FoundationOne® CDx (F1CDx) (Jun 2018–Jan 2023). For patients with multiple submitted biopsies of the same type, only a single sample with the highest sequencing quality metrics was included. For tissue/liquid concordance analysis, a convenience cohort of 5756 patients with both tissue and liquid biopsy results available was analyzed (Fig. 1A).

### MONSTAR-SCREEN cohort

SCRUM-Japan MONSTAR-SCREEN is anationwide cancer genome screening project aimed at prospectively assessing ctDNA in patients with advanced solid tumors other than lung cancer at 31 Japanese institutions (UMIN 000036749). Patients enrolled in MONSTAR-SCREEN from July 2019 to January 2021 were analyzed. The main eligibility criteria were the presence of histologically proven solid tumors; the presence of unresectable lesions; and at Eastern Cooperative Oncology Group performance status of 0–1. All patients provided written, informed consent prior to participating in this observational study. The study protocol was approved by the Institutional Review Board of each participating institution.

Tissue and blood samples were collected prior to the initiation of treatment. Blood samples were profiled using F1LCDx, and tissue samples were profiled using F1CDx. microsatellite instability (MSI) status was analyzed using F1CDx or a PCR-based MSI test kit (FALCO Biosystems) (Fig. 1B).

### Survival analysis

We assessed ORR, PFS, and overall survival (OS). Tumor response was assessed according to the guidelines of the Response Evaluation Criteria in Solid Tumors version 1.1. ORR was defined as the proportion of patients with the best overall response of complete or partial response (CR/PR). PFS was defined from the date of initiation of ICIs to the date of disease progression or death from any cause. OS was defined as the time from ICIs initiation to death from any cause. For this efficacy analysis, we used the bTMB≥14 cutoff, as it demonstrated the significant improvement of PFS afforded by atezolizumab compared with chemotherapy in an exploratory analysis of the BFAST trial, a randomized phase 3 trial for NSCLC [8].

Statistical comparisons of ORR were performed using Chi-square test or Fisher's exact test. PFS was estimated using the Kaplan–Meier method and compared according to biomarkers using Cox proportional hazards models and presented as HR with 95%CIs. Statistical significance was set at *p*<0.05. All statistical analyses were performed using the statistical program R, version 4.1.0 (The R Foundation for Statistical Computing, Vienna, Austria).

### Comprehensive genomic profiling of liquid and tissue biopsies

All CGP assays were performed in a Clinical Laboratory Improvement Amendment-certified, College of American Pathologists-accredited, New York State-approved laboratory (Foundation Medicine, Cambridge, MA).

Circulating cell free DNA was extracted from whole blood and analyzed using F1LCDx, a validated in vitro diagnostic device that targets 324 cancer-related genes (see Supplementary Table 8 for complete list of genes) [12]. The assay uses hybrid-capture technology and deep sequencing coverage to report single nucleotide variants, indels, genomic rearrangements, copy number amplifications and losses, and genomic signatures including bTMB, MSI, and tumor fraction (TF) which estimates the percentage of ctDNA in the cell-free DNA extracted from plasma. Foundation Medicine's ctDNA TF on F1LCDx is a composite algorithm prioritizing aneuploidy at higher levels to avoid germline signal and prioritizing variant allele frequency of canonical alterations at lower levels to maximize dynamic range, which merges two methods for estimation of TF (also see **Supplementary Methods**) [13,14].

Tissue biopsies were analyzed using F1CDx [15]. All samples forwarded for DNA extraction contained a minimum of 20% tumor nuclei. 50-1,000 ng of DNA are used for whole genome shotgun library construction and hybrid-capture of the same 324 cancer related genes as F1LCDx. See supplementary methods for assessment of TMB, microsatellite instability and TF.

## Results

### Detection of high blood mutational burden and microsatellite instability across cancer types

In a clinical cohort of 48,521 patients who underwent liquid biopsy in the course of routine clinical care (Figs. 1A), 5745 (11.8%) and 2850 (5.9%) had a bTMB≥10 and ≥ 14, respectively (Supplementary Table 1). Detection of high bTMB was associated with a higher estimated tumor fraction: the samples with bTMB≥10 had a median TF of 13.9% (IQR: 4.5-35.1%), while in the 42,776 samples with bTMB<10, the median TF was 1.4% (IQR: 0.0-6.5%).

**Fig. 2:**
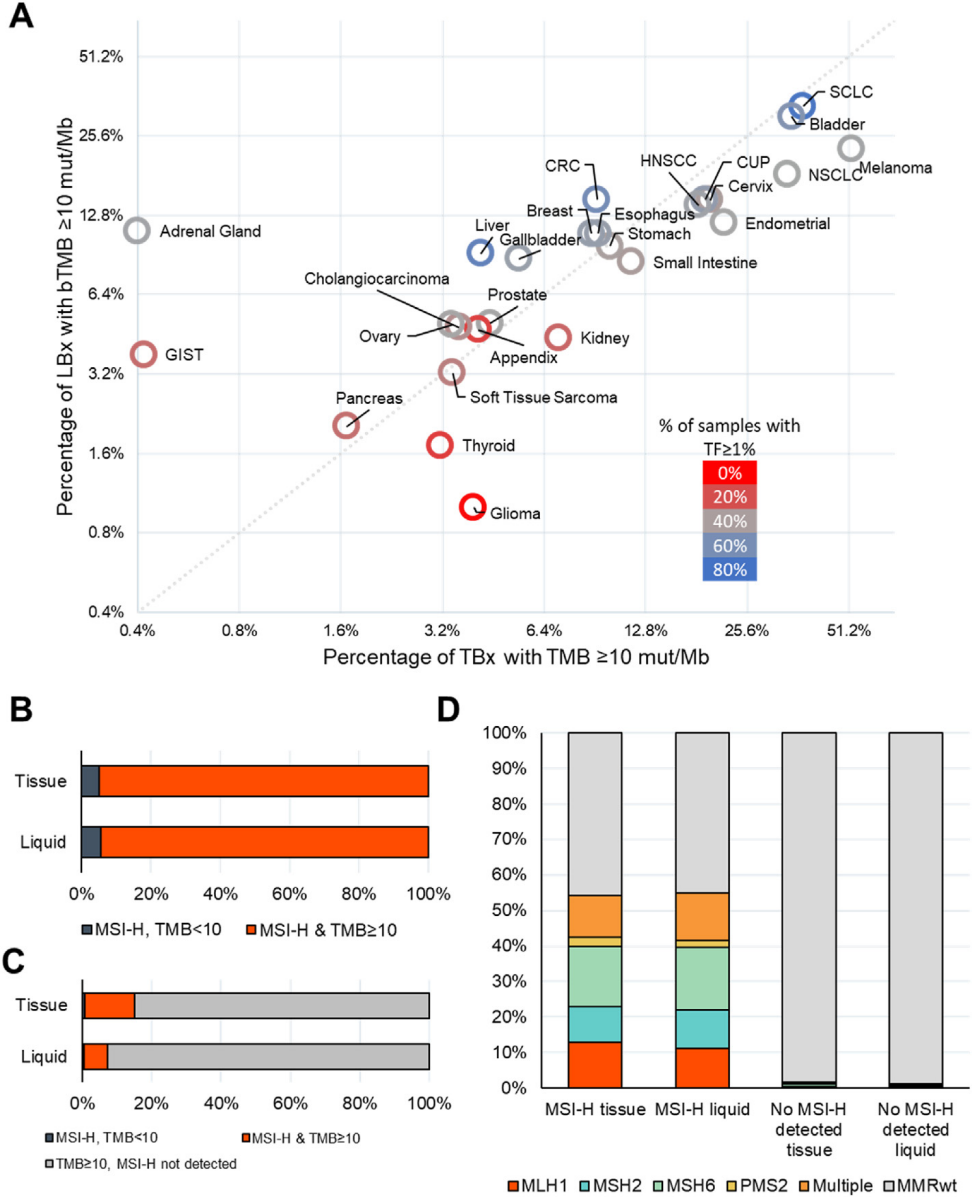
Detection of elevated tumor mutational burden and microsatellite instability in liquid biopsies. A) Prevalence of elevated bTMB ≥10 mut/Mb in liquid compared to prevalence of TMB ≥10 in tissue. Shade of bubble corresponds to ctDNA shed. B) Similarly to tissue biopsies, the majority of samples with high microsatellite instability (MSI-H) detected are bTMB ≥10 (95% of tissue, 94% of liquid biopsies). C) MSI-H accounts for 14.4% of TMB ≥10 tissue biopsies, and 6.9% of bTMB ≥10 liquid biopsies. D) Detection of MSI-H heavily overlapped with detection of loss of function mutations in a mismatch repair (MMR) genes in both tissue and liquid biopsies. CUP: carcinoma of unknown primary; CRC: colorectal cancer; GIST: gastrointestinal stromal tumor; HNSCC: head and neck cancer; NSCLC: non-small cell lung cancer; SCLC: small cell lung cancer; mut/Mb: mutations per megabase. (For interpretation of the references to color in this figure legend, the reader is referred to the Web version of this article.)

Cancer types with high rates of TMB-high tissue biopsies tended to have similarly high rates of bTMB-high liquid biopsies. Bladder, lung, and melanoma cancers had high frequencies of both TMB and bTMB≥10, in contrast to pancreatic and thyroid cancers and sarcomas (Fig. 2A, Supplementary Table 2). ctDNA shedding rates in different cancer types appeared to affect bTMB detection. In high-shedding cancer types, such as colorectal cancer (CRC, median TF 4%), small cell lung cancer (SCLC, 25.9%), and liver cancer (9.9%), bTMB≥10 was detected with an equal or greater frequency than TMB≥10 (CRC: 9% TMB≥10 versus 15% bTMB≥10; SCLC: 37% versus 33%; liver: 4% versus 9%). In contrast, cancer types with high rates of TMB≥10 and lower ctDNA shed like non-small cell lung cancer (NSCLC) and endometrial cancer (both with median TF 0.4%), bTMB≥10 was detected less frequently than TMB≥10 (NSCLC: 34% TMB≥10 versus 18% bTMB≥10; endometrial: 22% versus 14%).

### Detection of microsatellite instability across cancer types

High microsatellite instability (MSI-H) was detected in 423 (0.9%) liquid biopsies, compared to 2.4% of tissue biopsies. Among the 21,430 liquid biopsies with TF≥1%, 399 (1.9%) had MSI-H detected (Supplementary Table 1). For the majority of cancer types prevalence of MSI-H was similar between tissue biopsies and liquid biopsies with TF≥1% (Supplementary Fig. 1, Supplementary Table 3). The vast majority (399/423, 94%) of MSI-H liquid biopsies were also bTMB≥10, accounting for 399/5,745, 6.9%) of the samples with bTMB≥10 (Fig. 2B and C). Similarly to tissue samples, 232 (55%) of the liquid biopsies with MSI-H detected had a loss-of-function mutation in one or more of the mismatch repair (MMR) genes *MLH1, MSH2, MSH6*, or *PMS2* (Fig. 2D).

### Mutational landscape of samples with high and low bTMB

**Fig. 3:**
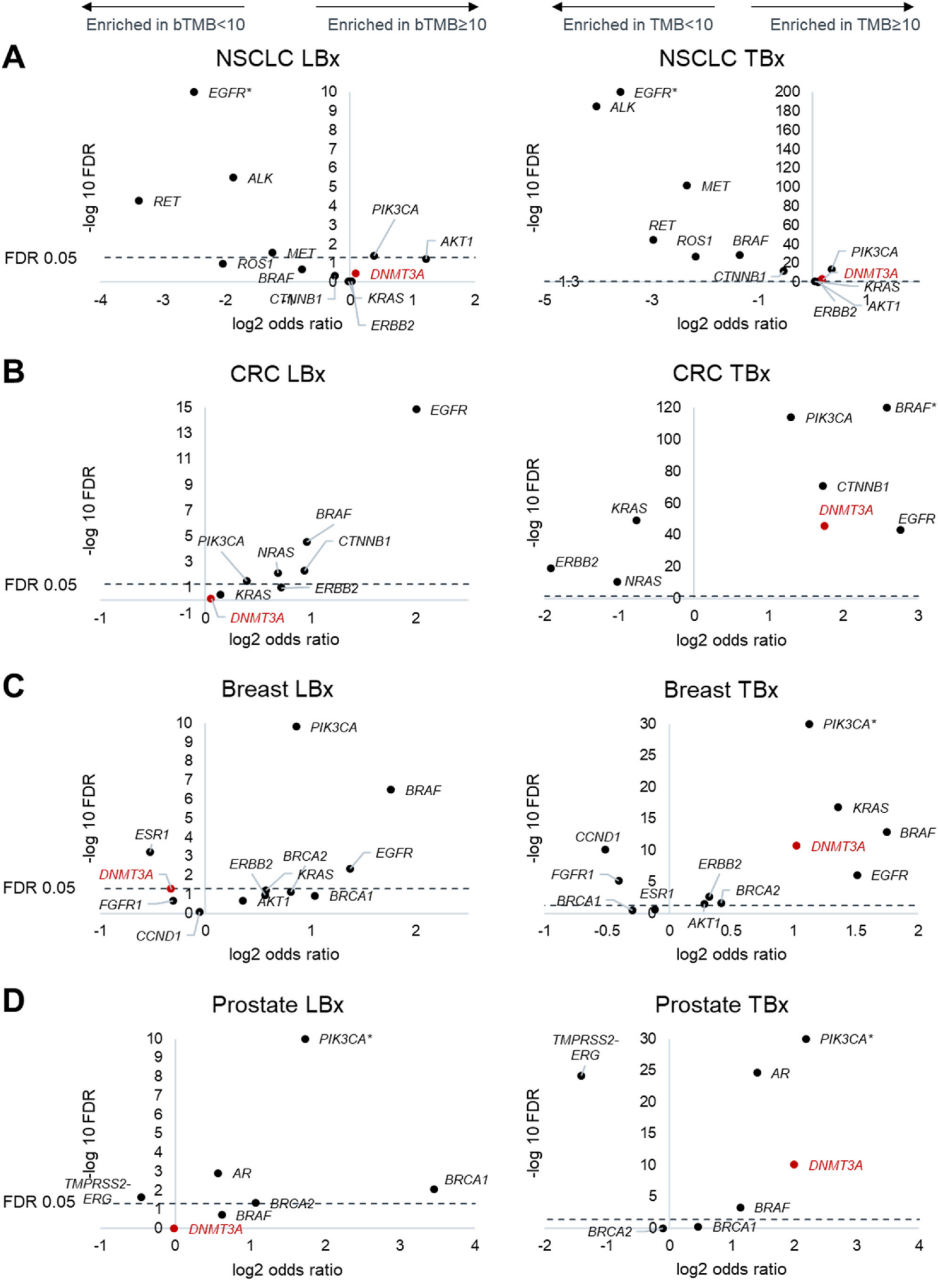
Liquid biopsies with TF of at least 1% were analyzed for enrichment of relevant driver variants in 4 cancer types. An analysis of tissue biopsies with TMB ≥10 versus <10 mut/Mb is provided on the right for comparison. A) NSCLC (N = 2782 bTMB<10 LBx versus 1743 bTMB ≥10 LBx; 41030 TMB<10 TBx versus 20786 TMB ≥10 TBx) B) CRC (N = 1948 versus 600; 35893 versus 3624) C) Breast cancer (N = 2426 versus 668; 29461 versus 2860) D) Prostate cancer (N = 3366 versus 414; 16573 versus 769) Dashed line demarcates FDR = 0.05. Genes marked with an asterisk were offscale along the y-axis and moved down into the plot. Note that the pathogenic variants analyzed here are excluded from TMB and bTMB calculations. *DNMT3A* (a common CH variant) is included in each plot in red. For breast and prostate cancer only *BRCA1/2* variants predicted to be germline were considered in the analysis. LBx: liquid biopsy, TBx: tissue biopsy, FDR: false discovery rate. (For interpretation of the references to color in this figure legend, the reader is referred to the Web version of this article.)

We examined the mutational landscape of relevant driver alterations in the four most representative cancer types in the liquid biopsy cohort. These pathogenic alterations are not counted into the TMB and bTMB biomarkers of this study. In NSCLC, liquid biopsies with detected *EGFR* driver mutations, *ALK, RET,* and *ROS1* fusions, and *MET* exon 14 skipping mutations tended to have bTMB<10, similarly to NSCLC tissue biopsies (Fig. 3A). In CRC, samples with *BRAF* V600E, and activating mutations in *PIK3CA, EGFR,* and *CTNNB1* were more likely to have TMB and bTMB≥10. In tissue biopsies, samples with *KRAS/NRAS* mutations, or *ERBB2* amplifications tended to have TMB<10, however liquid biopsies showed no such trends, potentially owing to a more complex acquired resistance landscape in these genes in liquid biopsies (Fig. 3B). In breast cancer, most oncogenic alterations were enriched in TMB and bTMB≥10 samples, with the notable exception of *ESR1*, consistent with hormone-receptor positive breast cancer having lower TMB than other subtypes (Fig. 3C). In prostate cancer, oncogenic alterations in *PIK3CA* and *AR* were enriched in TMB and bTMB≥10 samples, whereas *TMPRSS2-ERG* fusions were more likely to be found in TMB and bTMB<10 samples (Fig. 3D).

Of note, mutations in *DNMT3A*, which are implicated in driving clonal hematopoiesis (CH), are frequently detected in liquid biopsies. These mutations were not enriched in samples with bTMB≥10 in any of the four cancers examined. At the same time, *DNMT3A* mutations are enriched in tissue biopsies with TMB≥10. This is consistent with *DNMT3A* mutations appearing rarely in tissue biopsies, and tending to appear only in high TMB tissue samples, often as passenger disruptions of the gene, but appearing frequently in liquid biopsies regardless of tumor mutational burden (Fig. 3A-D). Blood samples with CH signal (*DNMT3A* mutations present) were not more likely to have bTMB≥10 detected.

### Concordance of blood and tissue TMB

**Fig. 4:**
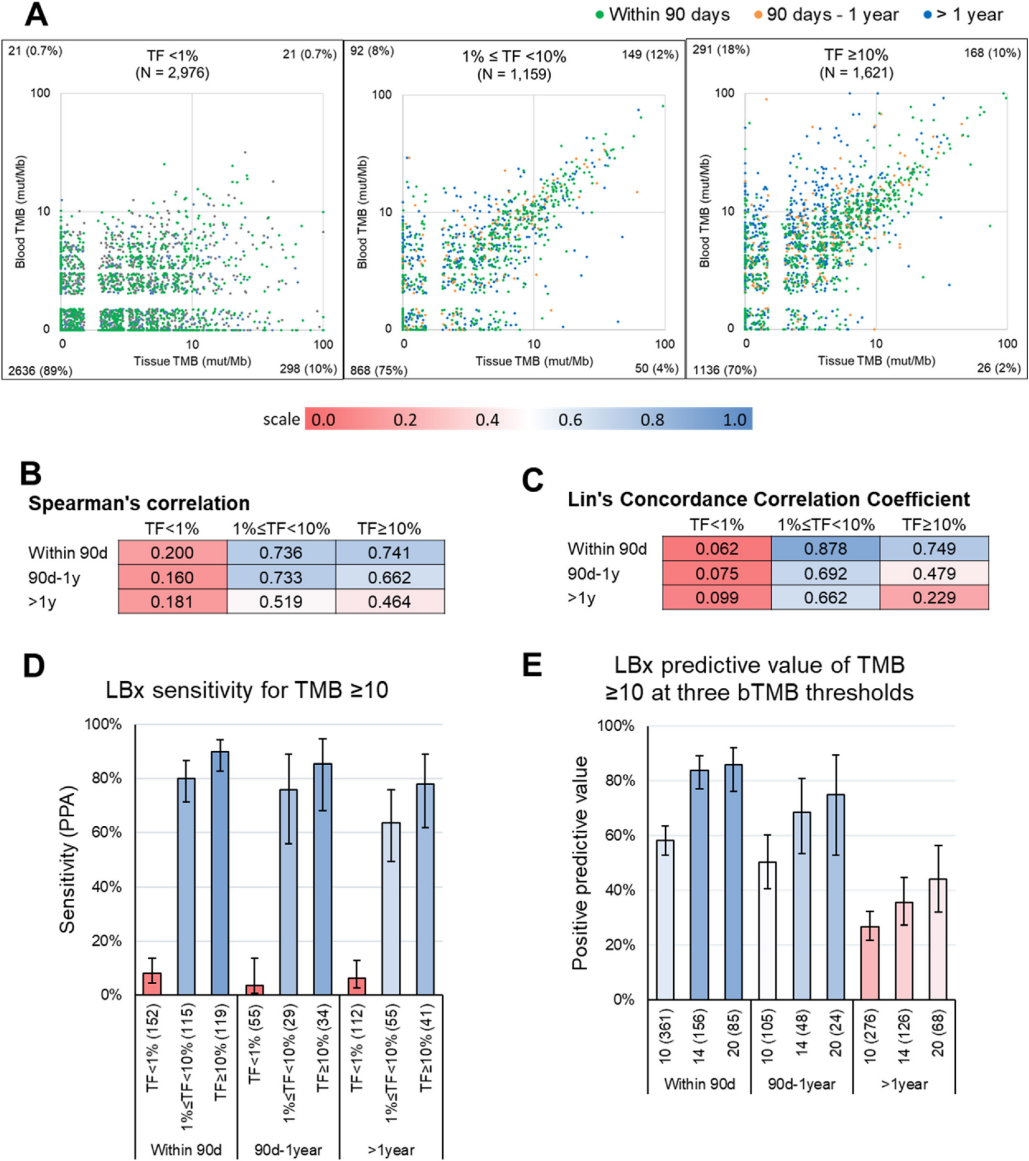
A) Scatterplots of tissue biopsy TMB and liquid biopsy bTMB from the same patient. Samples are grouped into 3 bins according to TF (<1%, 1-10% and ≥10%). Color denotes time separation between tissue and liquid specimen collection. Numbers in the corner of each plot denote how many points fall into each quadrant. B) Spearman's rank correlation for tissue/liquid pairs, organized by TF and collection date separation bins. C) Same as in (B) but Lin's concordance correlation coefficient values. D) Sensitivity of detection, as calculated by positive percent agreement (PPA) between tissue/liquid pairs, with tissue TMB ≥10 mut/Mb taken as standard. Error bars denote Wilson continuity-corrected 95% confidence intervals. Numbers in parentheses denote number of pairs with TMB ≥10 in the tissue biopsy. E) Positive predictive value (PPV) of liquid biopsy to predict TMB ≥10 when bTMB is ≥10, ≥14, or ≥20 mut/Mb. Error bars denote Wilson continuity-corrected 95% confidence intervals. Numbers in parentheses denote number of pairs with a liquid sample with a bTMB at or above the threshold of 10, 14, or 20 respectively. (For interpretation of the references to color in this figure legend, the reader is referred to the Web version of this article.)

We analyzed the concordance of bTMB and TMB in a cohort of 5756 patients who had both tissue and liquid genomic profiling results, with specimens collected no more than 90 days before tissue (median: liquid collected 205 days after tissue [IQR: 19-685 days]) (Fig. 4A, Supplementary Table 4). In samples with TF<1%, Spearman's correlation was much lower than for samples with TF between 1 and 10%, and with TF≥10% (0.200 vs 0.736 and 0.741 respectively, among pairs collected within 90 days of each other) (Fig. 4B). Lin's concordance correlation coefficient (CCC), which quantifies correlation with the expectation that the relationship will be 1:1, was 0.75 and 0.88 for the sets with TF≥10% and TF1-10%, but only 0.06 for the set with TF<1% (Fig. 4C). We confirmed this finding in 331 patients from the prospective MONSTAR-SCREEN cohort who had both tissue and liquid results available, with zero bTMB>10 true positives detected in the TF<1% subset, but improved concordance in the TF1-10% and elevated TF≥10% subsets (Supplementary Fig. 2).

Sensitivity of detection of TMB≥10 as measured by bTMB≥10 was 80% in samples with TF1-10%, and 90% in TF≥10%, but only 8% in samples with TF<1%. Sensitivity of detection was not strongly affected by increasing time between sample collection: for TF≥10% samples, sensitivity was 90% in pairs collected within 90 days of each other, 85% for pairs collected 90 days to 1 year, and 78% for pairs collected more than a year apart, but these differences were not significant (Fig. 4D).

Positive predictive value (PPV) of bTMB was assessed at three different thresholds: 10, 14 and 20mut/Mb. As expected, PPV rose at higher thresholds (Fig. 4E). The effect of collection time was more striking on PPV than on sensitivity. At bTMB threshold of 14 and 20, PPV was 84% and 86%, respectively, for pairs with specimens collected within 90 days of each other. The PPV at the same thresholds was only 36% and 44% for pairs with specimens collected more than one year apart (Fig. 4E). This suggests that high bTMB values in many of these pairs may not be a “false positive” but rather the result of increasing heterogeneity over time, leading to higher mutational burden, especially in liquid which can detect mutations accumulating in multiple clones and metastatic sites.

### Cases of high blood TMB discordance

We examined 121 discordant pairs where tissue detected TMB<10 but the liquid biopsy registered a bTMB of ≥14. The majority of these samples were from patients with CRC (48, 40%), breast cancer 28 (23%), and NSCLC (26, 21%) (Supplemental Fig. 3). Many of the liquid biopsies among these pairs had a dominant mutational signature. For CRC, 18/48 (38%) bore a mutational signature consistent with exposure to 5-fluorouracil (COSMIC SBS17), and 7 (15%) had MMR signature. In contrast, among the breast cancer pairs, 18/28 (64%) had a dominant APOBEC signature detected. MMR signatures were also detected among pairs from patients with endometrial and prostate cancers. Thus, the dominant signatures were associated with mutational processes or therapies for the particular cancer type, suggesting the high mutational burden detected in the liquid sample is indeed derived from the tumor and not a technical or analytical error.

### Concordance of blood and tissue MSI-H detection

**Fig. 5:**
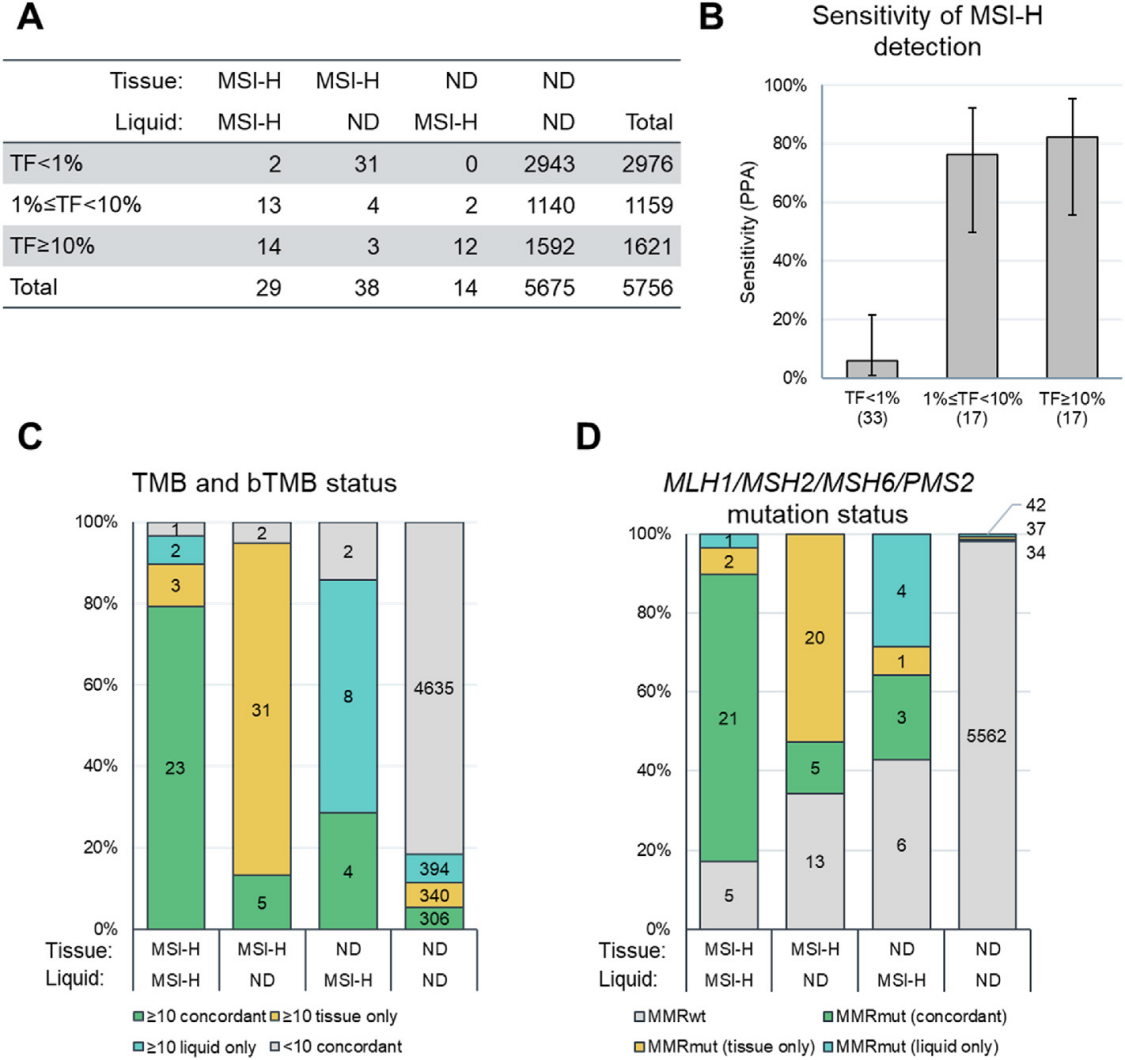
A) MSI-H detection in tissue and liquid biopsies among the 5756 pairs, broken down by TF bin. B) Liquid biopsy sensitivity of detection of MSI-H in tissue, as a function of TF. Numbers in parentheses are the number of pairs where tissue detected MSI-H. C) Association of MSI-H detection in tissue and liquid biopsy, and detection of TMB/bTMB ≥10 in the same sample. D) Association of MSI-H detection in tissue and liquid biopsy, and detection of variants in mismatch repair genes *MLH1, MSH2, MSH6,* or *PMS2*. ND: MSI-H not detected (or microsatellite stable); MMRmut: mutation in a mismatch repair gene.

We also examined the concordance of MSI-H detection in the patient-matched tissue/liquid biopsy pairs. Among the 5756 pairs, MSI-H was concordantly detected in 29 pairs, detected only in tissue in 38 pairs, and only in liquid in 14 pairs, for an overall sensitivity of detection of 29/67 (43%) (Fig. 5A). However, sensitivity was 76% and 82% in the TF1-10% and TF≥10% subsets, respectively (79% for TF≥1%), whereas in the TF<1% group sensitivity was only 6% (Fig. 5B). Pairs with concordantly detected MSI-H tended to have concordantly detected TMB and bTMB≥10 (Fig. 5C). In pairs with concordant detection of MSI-H, 24/29 (83%) had a mutation in a mismatch repair gene. In pairs with tissue-exclusive or liquid-exclusive MSI-H detection, 25/38 (66%) and 8/14 (57%) had an MMR mutation, respectively. All three of these groups had much higher rates of mutations in MMR genes than pairs where MSI-H was not detected by either platform, 113/5675 (2.0%) (Fig. 5D).

### Outcome on ICIs therapy in patients with elevated bTMB

Between July 2019 and February 2022, a total of 2190 patients were enrolled in MONSTAR-SCREEN. Of these, 459 patients were treated with ICIs without cytotoxic chemotherapy after enrollment and had an available bTMB score (Fig. 1B). Cancer types with a TMB≥10 frequency of ≥25% in Supplementary Table 2 were defined as ‘hypermutated type’ and were classified as hypermutated and non-hypermutated type. There were 133 hypermutated type (29%) and 326 non-hypermutated type (71%). The other baseline characteristics of the 459 patients are shown in Supplementary Table 5. Of 459 patients, 49 (11%) had tumors with bTMB≥14. The median period between blood collection and the start of ICIs was 0 days (range, 0–72 days), and none of the patients received any other treatment between blood collection and ICIs start. In this cohort, tissue analysis by F1CDx was performed for 127 patients, in whom TMB≥10 was confirmed in 23 patients (5%), including 9 with bTMB≥14. Twenty of the 49 patients with bTMB≥14 also had MSI-H detected by ctDNA analysis. Of 154 patients with tissue MSI status available, MSI-H was detected in 30 patients, including 22 with bTMB≥14. The median follow-up time was 9.2 months (range, 0.3–35.1 months) at the data cut-off (December 2022).

**Fig. 6:**
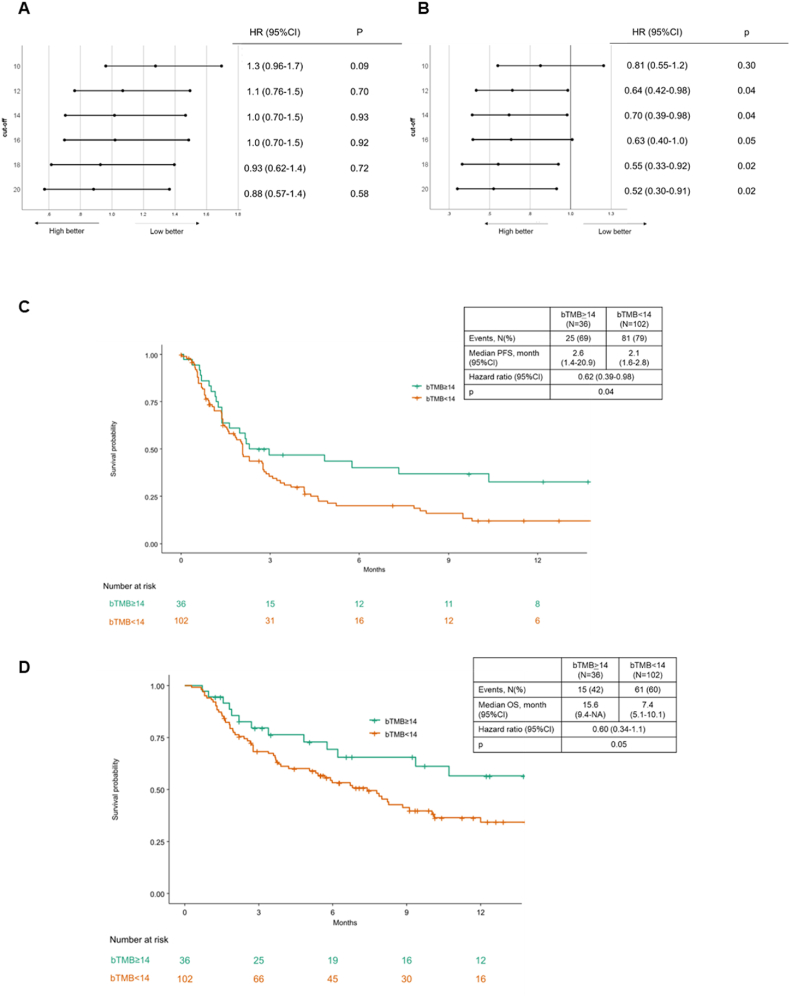
Efficacy of ICIs. A) Forest plots of hazard ratio for progression-free survival (PFS). Statistical tests (log-rank) were unadjusted for multiple comparisons and two-sided at the 0.05 significance level. The error bars show the 95%CIs. B) Forest plots of hazard ratio for PFS in patients with TF≥10%. Statistical tests (log-rank) were unadjusted for multiple comparisons and two-sided at the 0.05 significance level. The error bars show the 95%CIs. C) Kaplan–Meier analysis of the probability of PFS in bTMB≥14 with TF≥10% group as compared with bTMB<14 with TF≥10% group. Vertical lines denote patients who were censored. D) Kaplan–Meier analysis of the probability of OS in bTMB≥14 with TF≥10% group as compared with bTMB<14 with TF≥10% group. Vertical lines denote patients who were censored.

At the data cutoff, median PFS was 4.0 months (95%CI, 3.4-4.7) and median OS was 17.4 months (95%CI, 13.8-21.0) in the overall population. The hazard ratio (HR) for PFS monotonically improved with increasing bTMB cutoff (Fig. 6A). Given the high concordance between TMB and bTMB in tumors with elevated TF, we assessed the efficacy by bTMB and TF. Indeed, this tendency is particularly strong in patients in TF≥10% (Fig. 6B). The median PFS was 3.0 (95%CI, 1.8–19.4) and 4.1 (95%CI, 3.5–4.6) months in bTMB≥14 and < 14 groups, respectively (hazard ratio (HR, 1.02 [95%CI, 0.70–1.47]; p = 0.93) (Supplementary Fig. 4A). Patients with elevated TF≥10% had shorter PFS than patients with lower TF (Supplementary Table 6). Nevertheless, in this population with elevated TF≥10%, the median PFS in bTMB≥14 group was 2.6 months (95%CI, 1.4–20.9) with an estimated 6-month PFS of 40.1% (95%CI, 26.7–60.6) and estimated 12-month PFS of 32.7% (95%CI, 19.8–54.0), which was significantly longer than that in bTMB<14 group (HR, 0.62 [95%CI, 0.39–0.98]; p = 0.04) (Fig. 6C). Similarly, median OS was significantly longer in the bTMB≥14 group than in the bTMB<14 group in patients with elevated TF ≥10% (median, bTMB≥14: 15.6 months vs. bTMB<14: 7.4 months, HR, 0.60 [95%CI, 0.34-1.1]; p = 0.05) (Fig. 6D). The ORR was 22.4% (11/49, 95%CI, 12.9%–36.0%) in bTMB≥14 group and 19.4% (7/36, 95%CI, 9.4%–35.3%) in bTMB≥14 and TF>10% group.

In patients with MSI-H tumors, the PFS was not significanttly different between patients with MSI-H and MSI-L/MSS tumors (HR, 0.68 [95%CI, 0.38-1.2]; p = 0.20) (Supplementary Fig. 4B). For TMB, there was also no difference in PFS between TMB-H patients and TMB-L patients (HR, 0.83 [95%CI, 0.45–1.5]; p = 0.55) (Supplementary Fig. 4C). After adjusting the Cox-hazard model using the multivariate analysis findings, treatment line and bTMB≥14 were identified as the independent predictors for PFS (Supplementary Table 7).

Genomic alterations and treatment response were analyzed in patients with elevated TF≥10%. While bTMB score was not significantly different among patients with CR/PR, stable disease (SD), and progression disease (PD), copy number alterations (CNAs) were significantly more common in patients with PD than those with SD or CR/PR patients (p<0.05, Supplementary Figs. 4D and E).

## Discussion

We evaluated genomic ICIs biomarkers detected by CGP of tissue and ctDNA. To the best of our knowledge, this study is the largest study to compare ICIs biomarker detection in tissue versus blood. Moreover, we evaluate the ctDNA landscape in association with efficacy of ICIs in the prospective MONSTAR-SCREEN study, which is a nationwide profiling and monitoring project of ctDNA with advanced solid tumors.

This study compared frequencies of bTMB and TMB≥10 mut/Mb across >25 different solid tumor types. In contrast to previous reports [16,17], we did not observe overall higher prevalence of bTMB-high, even at the low threshold of bTMB≥10. Rather, the frequency of bTMB-high tended to be comparable or lower than the frequency of TMB-high in most cancer types. Detection of high bTMB was associated with ctDNA shed, and was rarely detected in samples with ctDNA fraction below 1%.

Concordance analysis in tissue and liquid biopsies taken from the same patient shows strong association between TMB and bTMB. High Lin's concordance indices for liquid biopsies with TF≥1% suggest that most pairs have close to a 1:1 correlation, with no systematic overestimation of TMB by bTMB. The majority of false negatives (bTMB<10 while TMB≥10) were attributable to low TF (<1%) in the liquid biopsy. Many of the significantly discordant positives (bTMB≥14 while TMB<10) occurred in high TF liquid biopsies, collected more than 3 months after the tissue biopsy, and often bearing signatures consistent with exposure to DNA-damaging therapies or cumulative mutation acquisition over time in a DNA repair deficient setting. High TF liquid biopsy can detect more heterogeneity in tumors undergoing rapid evolution. It is important to emphasize, however, that in the majority of pairs (even those with TF≥10% in the liquid specimen, and even those collected years later than the tissue specimen) still had a bTMB within 5 mut/Mb of the TMB.

Taken together, bTMB is a reliable indicator of the mutational burden in the tumor when TF is at least 1%. In patients with advanced disease with significant tumor heterogeneity and high ctDNA shed, bTMB may be higher than the TMB measured in any single metastatic lesion. It is an open question whether patients who have high bTMB via heterogeneity are equally likely to benefit from ICIs as patients with a high clonal TMB.

This prospective study evaluated the utility of bTMB as a biomarker of ICI therapy outcome. In the MONSTAR-SCREEN cohort, blood and tissue genetic alterations of 459 patients treated with ICIs were analyzed. The prevalence of bTMB-high (defined as having ≥14 mut/Mb in F1LCDx) was 11% across solid tumor types. The blood samples were taken just before the initiation of ICIs, which may depict the tumor genomic landscape more accurately than archival tissue.

The ability of bTMB to represent true TMB is dependent on the ctDNA fraction. However, ctDNA fraction is a blood-based measure of disease burden and has prognostic implications of its own [ [14,18]. Levels of ctDNA shed is thus both an important quality metric and a prognostic biomarker. In the subset of patients with TF≥10% receiving ICIs, PFS and OS were significantly longer in patients with bTMB≥14 (p = 0.04 and p = 0.05). Elevated tumor fraction is a biomarker that is currently reported on some commercial liquid biopsy reports, with ongoing efforts to maximize the limits of detection in order to fully capture the potential utility described in this report.

Importantly, when bTMB is under-detected due to low ctDNA shed, such patients may still have durable responses on ICIs. A negative liquid biopsy may need to be confirmed with tissue-based NGS to evaluate for elevated TMB potentially missed in ctDNA, as is standard for patients with negative liquid biopsy results. Although recent prospective trials evaluating the efficacy of ICIs for patients with elevated bTMB, B-F1RST and CheckMate-848, failed to show the benefit of ICIs [9,19], these trials did not investigate efficacy according to TF. The potential of ICIs for elevated bTMB tumors with high ctDNA shed needs to be further investigated.

Genomic alterations and treatment efficacy were analyzed. Previous studies have shown that *PTEN* loss, *JAK1/2* mutation, and CNAs leading to loss of intact interferon-γ signaling were previously noted resistant to treatment with ICIs [[20], [21], [22], [23], [24]]. In particular, CNAs contribute to the initiation of cancer by mediating overexpression of oncogenes and to the development of cancer therapy resistance by increasing the expression of genes whose action diminishes the efficacy of anti-cancer drugs [25]. In our analysis, the number of CNAs was significantly higher in PD patients. However, it is also likely that detection of CNAs is associated with higher TF and thus higher disease burden.

It is important to note the limitations of the present study. First, the MONSTAR-SCREEN cohort was a limited sample size and follow-up time. Second, this cohort includes various cancer types, many with limited prior data on reported ICIs efficacy regardless of TMB score. Although the efficacy of ICIs was similar between ‘hypermutated’ and ‘non-hypermutated’ cancer types, a larger cohort is needed to confirm that bTMB is associated with the ICIs efficacy regardless of cancer types. Third, we used a targeted gene panel sequencing for analysis. Although these gene panel analyses have been reported to be highly correlated with whole-exome sequences, this may limit the interpretation of the results. Fourth, we did not analyze PD-L1 expression which is an important biomarker associated with the benefits of ICIs. Other biomarkers such as MMR and TMB could not be evaluated in all patients because tissue-based genomic and IHC results were not available for all cases. Nevertheless, these data indicate that blood-based NGS represents a less invasive diagnostic tool that should be made available to all patients, in particular those who are unable to provide tissue samples or those with insufficient tissue for tissue-based NGS.

In conclusion, this study provides evidence that bTMB can serve as a potential biomarker for predicting the efficacy of ICIs, and intriguingly may be more clinically useful in patients with advanced, aggressive, DNA shedding disease. Prospective investigations in larger cohorts are needed to confirm precise biomarkers of ICIs and to clarify clinical utility of bTMB for identifying high TMB tumors that may benefit from ICIs.

## Declaration of competing interest

The authors declare the following financial interests/personal relationships which may be considered as potential competing interests: Takayuki Yoshino reports a relationship with Taiho Pharmaceutical Co Ltd that includes: funding grants and speaking and lecture fees. Takayuki Yoshno reports a relationship with Chugai Pharmaceutical Co Ltd that includes: funding grants and speaking and lecture fees. Takayuki Yoshino reports a relationship with Eli Lilly and Company that includes: speaking and lecture fees. Takayuki Yoshino reports a relationship with Merck Biopharma Deutschland that includes: speaking and lecture fees. Takayuki Yoshino reports a relationship with Bayer Yakuhin Kabushiki Kaisha that includes: speaking and lecture fees. Takayuki Yoshino reports a relationship with Ono Pharmaceutical Co Ltd that includes: funding grants and speaking and lecture fees. Takayuki Yoshino reports a relationship with MSD that includes: funding grants and speaking and lecture fees. Takayuki Yoshino reports a relationship with Sanofi that includes: funding grants. Takayuki Yoshino reports a relationship with Daiichi Sankyo Inc that includes: funding grants. Takayuki Yoshino reports a relationship with Parexel International Corporation that includes: funding grants. Takayuki Yoshino reports a relationship with Pfizer Japan Inc Nagoya Factory Distribution Center that includes: funding grants. Takayuki Yoshino reports a relationship with Amgen Inc that includes: funding grants. Takayuki Yoshino reports a relationship with Genomedia that includes: funding grants. Takayuki Yoshino reports a relationship with Sysmex Corp that includes: funding grants. Takayuki Yoshino reports a relationship with Nippon Boehringer Ingelheim Co Ltd that includes: funding grants.
