# Supplementary material for: Validity and utility of blood tumor mutational burden (bTMB) is dependent on circulating tumor DNA (ctDNA) shed: SCRUM-Japan MONSTAR-SCREEN
Source: J Liq Biopsy. 2023 Aug 10;1:100003. doi: 10.1016/j.jlb.2023.100003 (PMC11863975; doi:10.1016/j.jlb.2023.100003)
Supplement: Multimedia component 1 [file mmc1.docx]

**SUPPLEMENTARY METHODS**

*Assessment of tumor mutational burden, microsatellite instability, and other mutational signatures*

Blood tumor mutational burden (bTMB) was measured by counting synonymous and non-synonymous short variants present at ≥0.5% variant allele frequency (VAF). Known and likely pathogenic variants were filtered out. Potential germline variants were filtered out using published databases of known germline polymorphisms including dbSNP, gnomAD, and ExAC, as well as by potential germline status prediction via a validated somatic-germline/zygosity (SGZ) algorithm [1]. The resulting mutation number was divided by the coding region (approximately 0.8 megabases). The ratio is reported in units of mutations per megabase (mut/Mb). TMB in tissue was calculated in a similar way, with a ≥5% VAF threshold applied to variants.

MSI status was measured based on ~2000 repetitive loci to determine repeat lengths present in the sample. A locus containing a repeat length present in an internal database generated using >3000 clinical samples was considered 'unstable'. An MSI indicator is generated by calculating the fraction of unstable loci, considering only those loci that achieve adequate coverage for consideration for the sample. Samples with >0.5% unstable loci are considered to be MSI-High [2,3].

In Figure 2 and Supplementary Figure 3, p values were adjusted using the Benjamini-Hochberg correction over all 324 genes. In Figure 3 and Supplementary Figure 5, jitter was added to points to avoid overplotting. In Figure 3C, differences between bTMB and TMB were capped at 50 and -50. In Supplementary Figure 6, mutational signatures were calculated using methods previously described [4], in samples with at least 10 assessable alterations and a successful SGZ call. Signatures were considered dominant if the maximum signature score was >0.4.

**References**

1. Sun JX, He Y, Sanford E et al. A computational approach to distinguish somatic vs. germline origin of genomic alterations from deep sequencing of cancer specimens without a matched normal. PLOS Comput Biol 2018; 14: e 1005965. doi; 10.1371/journal.pcbi.1005965, PMID 29415004.
2. Milbury CA, Creeden J, Yip WK et al. Clinical and analytical validation of FoundationOne®CDx, a comprehensive genomic profiling assay for solid tumors. PLOS ONE 2022; 17: e0264138. doi: 10.1371/journal.pone.0264138, PMID: 35294956.
3. Woodhouse R, Li M, Hughes J, Delfosse D, Skoletsky J, Ma P et al. Clinical and analytical validation of FoundationOne Liquid CDx, a novel 324-Gene cfDNA-based comprehensive genomic profiling assay for cancers of solid tumor origin. PLOS ONE 2020; 15: e0237802. doi: [10.1371/journal.pone.0237802](https://doi.org/10.1371/journal.pone.0237802), PMID [32976510](http://www.ncbi.nlm.nih.gov/pubmed/32976510).
4. Zehir A, Benayed R, Shah RH, Syed A, Middha S, Kim HR et al. Mutational landscape of metastatic cancer revealed from prospective clinical sequencing of 10,000 patients. Nat Med 2017; 23: 703-713. doi:10. 1038/nm.4333, PMID 28481359.
