# Supplementary material for: Validity and utility of blood tumor mutational burden (bTMB) is dependent on circulating tumor DNA (ctDNA) shed: SCRUM-Japan MONSTAR-SCREEN
Source: J Liq Biopsy. 2023 Aug 10;1:100003. doi: 10.1016/j.jlb.2023.100003 (PMC11863975; doi:10.1016/j.jlb.2023.100003)
Supplement: Multimedia component 2 [file mmc2.docx]

**Supplementary Figure 1: ctDNA shed association with detection of MSI-H**

**
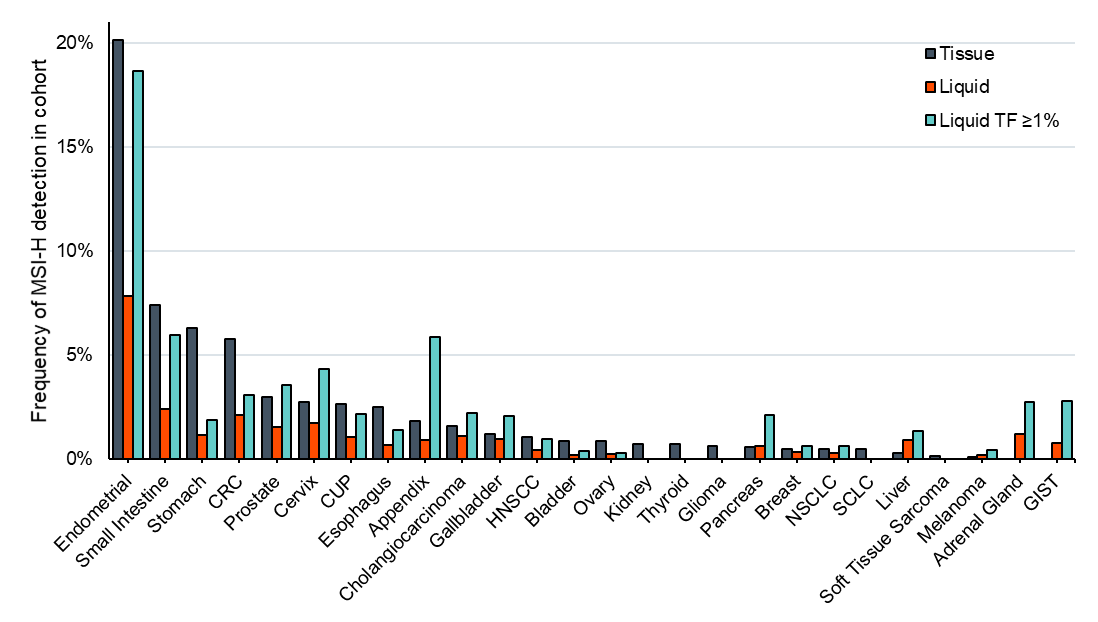
**

TF: tumor fraction, MSI-H: microsatellite Instability high, CRC: colorectal cancer, CUP: carcinoma of unknown primary, HNSCC: head and neck squamous cell carcinoma, NSCLC: non-small cell lung cancer, SCLC: small cell lung cancer, GIST: gastrointestinal stromal tumor

**Supplementary Figure 2: Effects of time on mutational burden concordance**

**
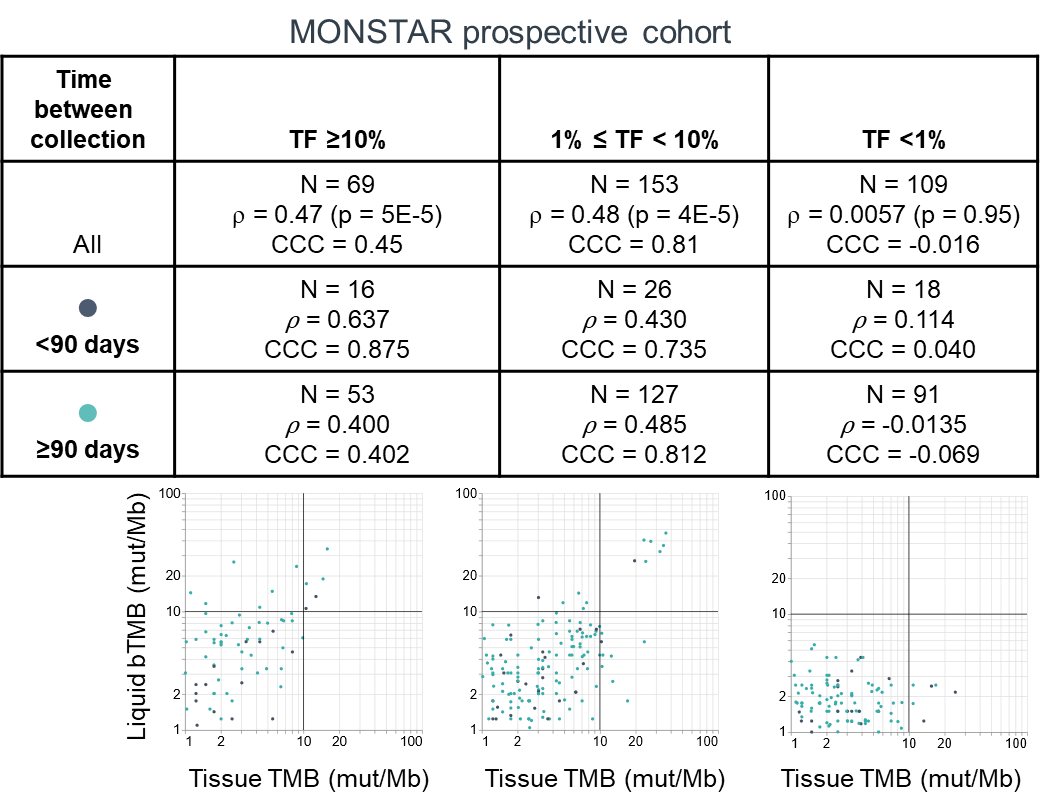
**Concordance of bTMB and TMB in liquid and tissue biopsies collected from the same patients with any time interval. Pairs are grouped according to level of ctDNA. Spearman’s rank correlation coefficient (rho) and Lin’s Concordance Correlation Coefficient (CCC) with a 95% confidence interval are provided for each group. For display purposes, TMB and bTMB values of 0 were set to 1 on these plots and jitter was added to alleviate overplotting. Pairs collected <90 days apart in dark blue, pairs collected ≥90 days apart in light blue.

TF: tumor fraction, bTMB: blood tumor mutational burden, TMB: tumor mutational burden

**Supplementary Figure 3: Analysis of genomic signatures in discordant high bTMB pairs**

**
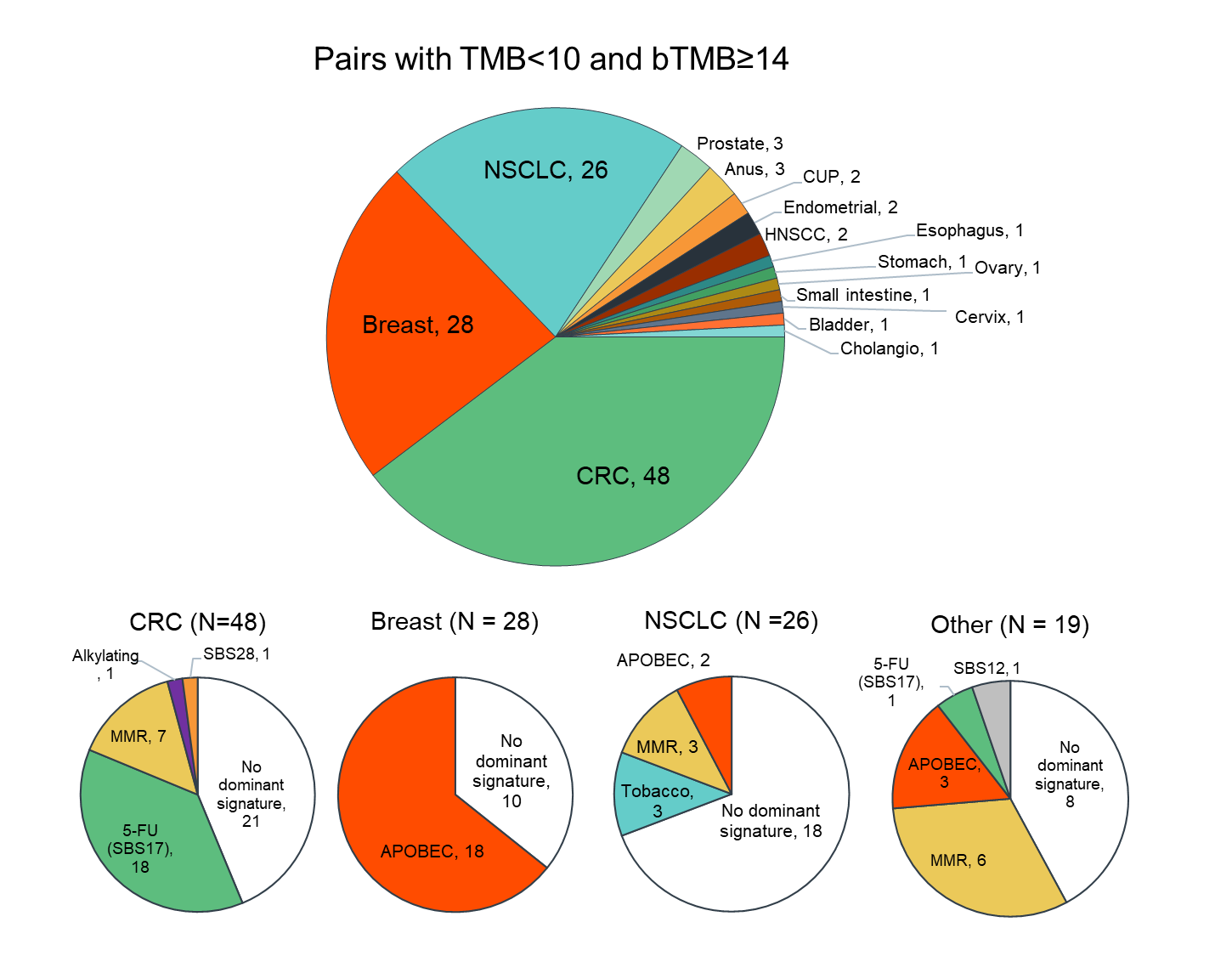
**

Analysis of tissue and liquid pairs where the tissue biopsy detected TMB <10 and the liquid biopsy detected bTMB ≥14. The top pie chart shows the cancer types predominating among these discordant pairs. The pie charts below show dominant mutational signatures that were detected in the liquid biopsies of these pairs.

TMB: tumor mutational burden, bTMB: blood tumor mutational burden, NSCLC: non-small cell lung cancer, CRC: colorectal cancer, CUP: carcinoma of unknown primary, HNSCC: head and neck squamous cell carcinoma, MMR: mismatch repair

**Supplementary Figure 4: Outcome on ICI therapy**

**A**

**
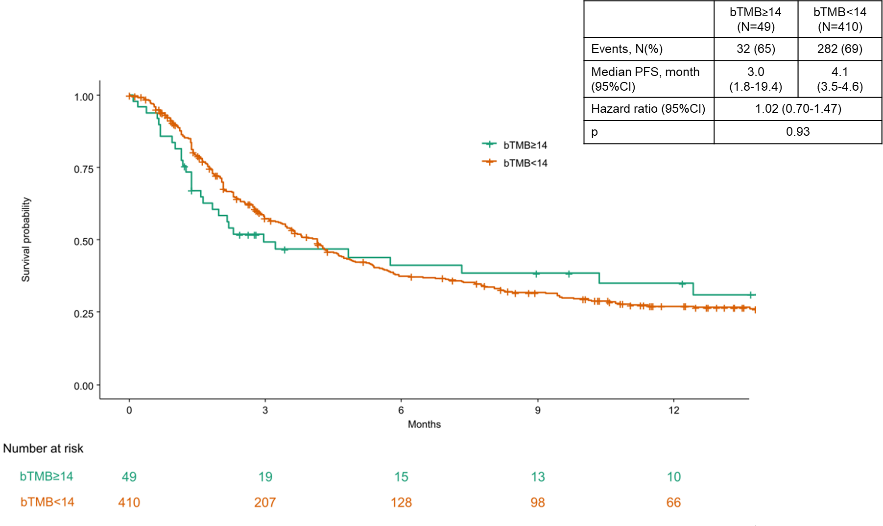
**

**B**

**
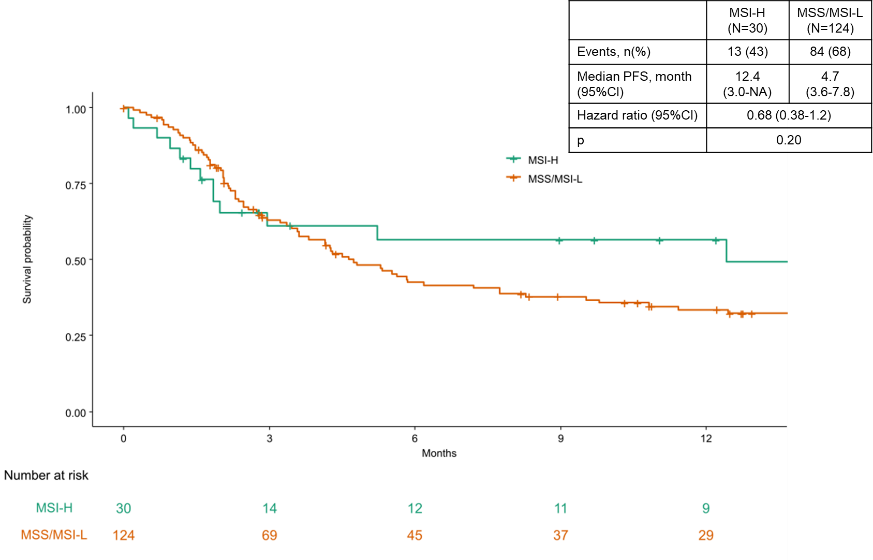
**

**C**

**
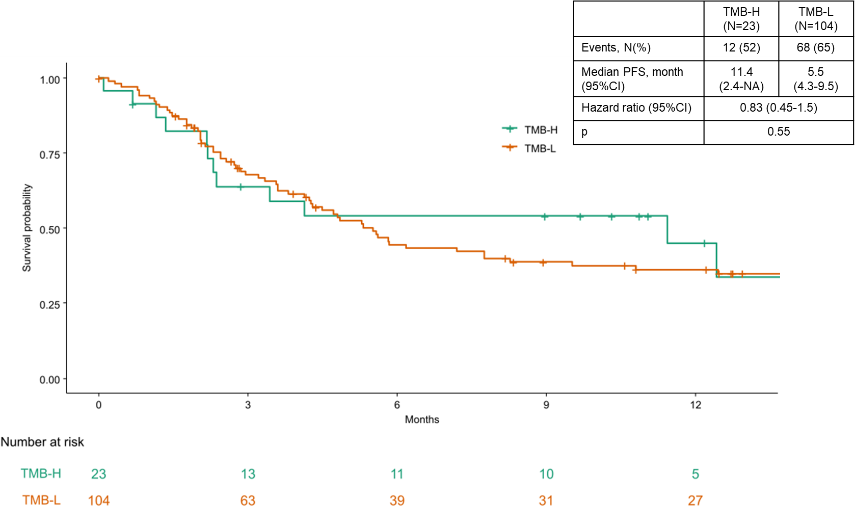
**

**
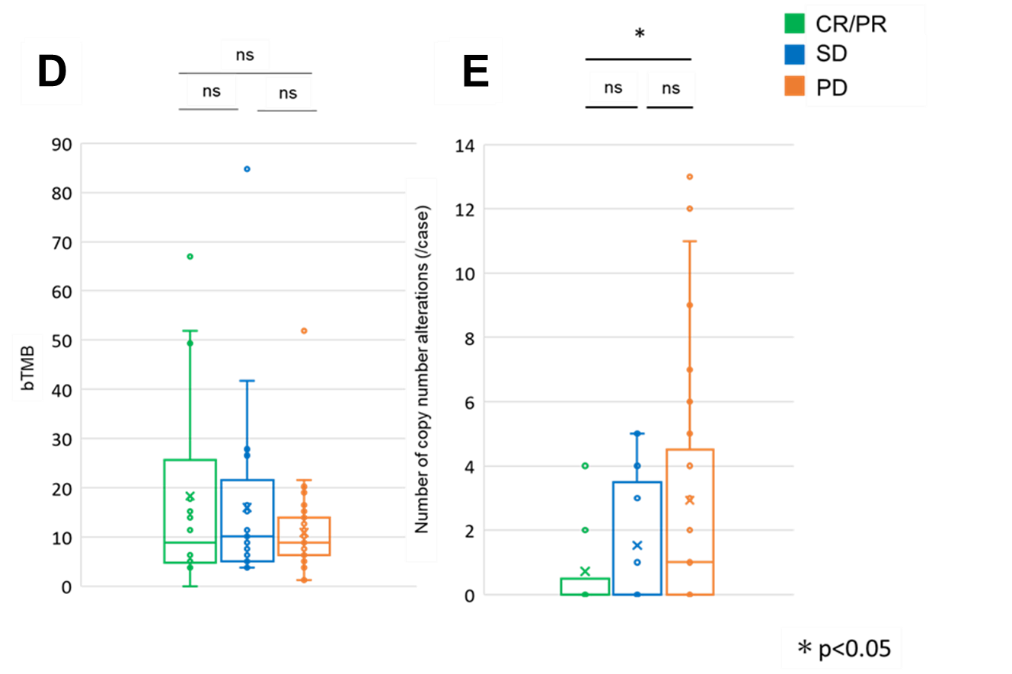
**

1. Kaplan–Meier analysis of the probability of progression-free survival (PFS) in the bTMB ≥14 patients as compared with the bTMB <14 patients. Vertical lines denote patients who were censored.
2. Kaplan–Meier analysis of the probability of PFS in the MSI-H patients as compared with the MSS/MSI-L patients. Vertical lines denote patients who were censored.
3. Kaplan–Meier analysis of the probability of PFS in the TMB-H patients as compared with the TMB-L patients. Vertical lines denote patients who were censored.
4. Comparison of bTMB between patients with complete response (CR) or partial response (PR), stable disease (SD), and those with progressive disease (PD). No significant difference between the groups. The boxes represent 25th–75th percentiles; center lines indicate the median; whiskers extend to the maximum and minimum values within 1.5× of the interquartile range; and dots indicate outliers.
5. Comparison of number of copy number alterations between patients with CR or PR, SD, and PD. Number of copy number alterations of CR/PR patients was significantly lower than PD patients (two-sided P <0.05, Mann–Whitney U-test). The boxes represent 25th–75th percentiles; center lines indicate the median; whiskers extend to the maximum and minimum values within 1.5× of the interquartile range; and dots indicate outliers.
