# Supplementary material for: Validity and utility of blood tumor mutational burden (bTMB) is dependent on circulating tumor DNA (ctDNA) shed: SCRUM-Japan MONSTAR-SCREEN
Source: J Liq Biopsy. 2023 Aug 10;1:100003. doi: 10.1016/j.jlb.2023.100003 (PMC11863975; doi:10.1016/j.jlb.2023.100003)
Supplement: Multimedia component 3 [file mmc3.docx]

**Supplementary Table 1: Prevalence of ICIs biomarkers in tissue and liquid biopsies**

Immunotherapy biomarkers in entire clinical cohort analyzed. Mutational burden in units of mutations per megabase (mut/Mb).

| **ICIs Biomarker** | **Tissue** | **Liquid, all** | **Liquid TF ≥ 1%** | **Liquid TF ≥ 10%** |
| --- | --- | --- | --- | --- |
| N | 313,296 | 48,521 | 21,430 | 12,518 |
| Mutational burden ≥10 mut/Mb | 50,146 (16%) | 5,745 (12%) | 5,309 (25%) | 3,353 (27%) |
| Mutational burden ≥14 mut/Mb | 29,497 (9%) | 2,850 (6%) | 2,709 (13%) | 1,726 (14%) |
| Mutational burden ≥20 mut/Mb | 19,939 (6%) | 1,701 (4%) | 1,633 (8%) | 1,068 (9%) |
| MSI-H | 7, 648 (2.4%) | 423 (0.9%) | 399 (1.9%) | 275 (2.2%) |

ICIs: immune checkpoint inhibitors, TF: tumor fraction, MSI-H: microsatellite Instability high

**Supplementary Table 2: bTMB and TMB levels across cancer types**

| **Cancer type** | **Liquid, N** | **Median TF**  **[IQR] (%)** | **TF ≥1% (%)** | **TF ≥10% (%)** | **bTMB ≥10 (%)** | **bTMB ≥14 (%)** | **bTMB ≥20 (%)** | **Tissue, N** | **TMB ≥10 (%)** | **TMB ≥14 (%)** | **TMB ≥20 (%)** |
| --- | --- | --- | --- | --- | --- | --- | --- | --- | --- | --- | --- |
| All | 48521 | 0.4 [0-10.7] | 44.2 | 25.8 | 11.8 | 5.9% | 3.5% | 313296 | 16.0% | 9.4% | 6.4% |
| NSCLC | 10690 | 0.4 [0-6.0] | 42.3 | 18.6 | 18.2 | 8.7% | 4.5% | 61816 | 33.6% | 17.5% | 9.5% |
| Prostate | 8990 | 0.1 [0-16.6] | 42.0 | 30.6 | 5.1 | 2.4% | 1.9% | 17342 | 4.4% | 3.3% | 2.8% |
| Breast | 6395 | 0.8 [0-14.1] | 48.4 | 30.4 | 10.9 | 5.6% | 3.5% | 32321 | 8.8% | 4.4% | 2.7% |
| CRC | 4166 | 3.8 [0.1-26.5] | 61.2 | 41.1 | 14.7 | 7.0% | 4.2% | 39517 | 9.2% | 6.5% | 6.0% |
| Pancreas | 3495 | 0 [0-1.5] | 28.6 | 12.4 | 2.1 | 0.9% | 0.7% | 21096 | 1.7% | 1.0% | 0.6% |
| CUP | 2327 | 1.0 [0-14.8] | 50.1 | 31.2 | 14.7 | 7.8% | 4.9% | 13875 | 19.2% | 11.8% | 8.3% |
| Cholangiocarcinoma | 1091 | 0.3 [0-9.2] | 41.8 | 24.4 | 4.8 | 2.0% | 1.5% | 6806 | 3.6% | 2.0% | 1.4% |
| Ovary | 922 | 0 [0-4.4] | 35.9 | 15.7 | 5.0 | 1.4% | 0.3% | 18953 | 3.4% | 1.1% | 0.8% |
| Esophagus | 735 | 0.8 [0-14.6] | 48.7 | 30.5 | 10.9 | 3.8% | 1.8% | 9434 | 9.3% | 3.6% | 2.5% |
| Kidney | 551 | 0 [0-1.2] | 26.3 | 11.8 | 4.4 | 2.0% | 1.3% | 5256 | 7.1% | 3.6% | 2.2% |
| Melanoma | 530 | 0.3 [0-12.6] | 43.4 | 28.1 | 22.8 | 16.4% | 13.2% | 9215 | 52.2% | 40.9% | 32.5% |
| Stomach | 518 | 0.2 [0-10.9] | 41.1 | 25.7 | 9.7 | 3.5% | 2.3% | 5776 | 10.0% | 6.6% | 5.6% |
| Bladder | 505 | 1.7 [0-13.4] | 54.5 | 30.1 | 29.9 | 16.4% | 9.3% | 8211 | 34.6% | 19.6% | 12.0% |
| HNSCC | 473 | 0.6 [0-12.6] | 45.0 | 28.5 | 14.2 | 8.7% | 5.3% | 5692 | 18.3% | 10.1% | 6.8% |
| Endometrial | 459 | 0.4 [0-6.6] | 42.0 | 20.9 | 12.2 | 9.4% | 8.1% | 10430 | 21.9% | 17.2% | 13.0% |
| Thyroid | 238 | 0 [0-0] | 16.0 | 5.5 | 1.7 | 0.8% | 0.4% | 2621 | 3.2% | 1.2% | 0.5% |
| Soft Tissue Sarcoma | 222 | 0 [0-3.9] | 33.8 | 18.9 | 3.2 | 2.3% | 2.3% | 671 | 3.4% | 2.7% | 2.4% |
| Liver | 220 | 9.9 [0.3-23.9] | 67.7 | 49.5 | 9.1 | 3.6% | 2.7% | 1468 | 4.2% | 1.2% | 0.9% |
| SCLC | 216 | 25.9 [1.5-64.9] | 77.8 | 61.1 | 33.3 | 13.9% | 6.0% | 2877 | 37.4% | 14.1% | 5.2% |
| Gallbladder | 206 | 0.8 [0-9.0] | 46.6 | 23.8 | 8.7 | 3.4% | 1.5% | 2059 | 5.4% | 2.2% | 1.7% |
| Cervix | 172 | 0.4 [0-8.5] | 40.1 | 22.1 | 15.1 | 9.9% | 7.0% | 2973 | 19.9% | 10.9% | 6.9% |
| Adrenal Gland | 166 | 0 [0-45.2] | 44.0 | 41.0 | 11.4 | 4.2% | 1.8% | 89 | 0.0% | 0.0% | 0.0% |
| Small Intestine | 166 | 0.4 [0-7.3] | 40.4 | 20.5 | 8.4 | 3.0% | 3.0% | 2094 | 11.6% | 7.6% | 6.6% |
| GIST | 133 | 0 [0-1.2] | 27.1 | 15.0 | 3.8 | 0.8% | 0.8% | 1434 | 0.4% | 0.3% | 0.3% |
| Appendix | 107 | 0 [0-0] | 15.9 | 5.6 | 4.7 | 1.9% | 0.9% | 1200 | 4.1% | 1.8% | 1.2% |
| Glioma | 102 | 0 [0-0] | 2.9 | 0.0 | 1.0 | 0.0% | 0.0% | 7105 | 4.0% | 3.2% | 2.9% |
| Other | 4726 | 0.5 [0-8.6] | 44.8 | 23.0 | 16.5 | 8.7% | 4.7% | 22965 | 12.5% | 8.7% | 7.0% |

bTMB: blood tumor mutational burden, TMB: tumor mutational burden, TF: tumor fraction, NSCLC: non-small cell lung cancer, CRC: colorectal cancer, CUP: carcinoma of unknown primary, HNSCC: head and neck squamous cell carcinoma, SCLC: small cell lung cancer, GIST: gastrointestinal stromal tumor

**Supplementary Table 3: Prevalence of MSI-H across cancer types**

| **Cancer Type** | **Tissue** | **Liquid** | **Liquid TF ≥1%** |
| --- | --- | --- | --- |
| Endometrial | 20.2% | 7.8% | 18.7% |
| Small Intestine | 7.4% | 2.4% | 6.0% |
| Stomach | 6.3% | 1.2% | 1.9% |
| CRC | 5.8% | 2.1% | 3.1% |
| Prostate | 3.0% | 1.5% | 3.5% |
| Cervix | 2.8% | 1.7% | 4.3% |
| CUP | 2.7% | 1.1% | 2.1% |
| Esophagus | 2.5% | 0.7% | 1.4% |
| Appendix | 1.8% | 0.9% | 5.9% |
| Cholangiocarcinoma | 1.6% | 1.1% | 2.2% |
| Gallbladder | 1.2% | 1.0% | 2.1% |
| HNSCC | 1.1% | 0.4% | 0.9% |
| Bladder | 0.9% | 0.2% | 0.4% |
| Ovary | 0.8% | 0.2% | 0.3% |
| Kidney | 0.7% | 0.0% | 0.0% |
| Thyroid | 0.7% | 0.0% | 0.0% |
| Glioma | 0.6% | 0.0% | 0.0% |
| Pancreas | 0.6% | 0.6% | 2.1% |
| Breast | 0.5% | 0.3% | 0.6% |
| NSCLC | 0.5% | 0.3% | 0.6% |
| SCLC | 0.5% | 0.0% | 0.0% |
| Liver | 0.3% | 0.9% | 1.3% |
| Soft Tissue Sarcoma | 0.1% | 0.0% | 0.0% |
| Melanoma | 0.1% | 0.2% | 0.4% |
| Adrenal Gland | 0.0% | 1.2% | 2.7% |
| GIST | 0.0% | 0.8% | 2.8% |

MSI-H: microsatellite Instability high, TF: tumor fraction, CRC: colorectal cancer, CUP: carcinoma of unknown primary, HNSCC: head and neck squamous cell carcinoma, NSCLC: non-small cell lung cancer, SCLC: small cell lung cancer, GIST: gastrointestinal stromal tumor

**Supplementary Table 4: Paired tissue/liquid cohort cancer types**

| **Specimen Collection** | **Cancer Type** | **TF<1%** | **1%≤TF<10%** | **TF≥10%** | **Total** |
| --- | --- | --- | --- | --- | --- |
| **Total** | **All** | 2976 | 1159 | 1621 | 5756 |
| **Within 90days** | **All** | 1228 | 566 | 686 | 2480 |
| **90days-1year** | **All** | 531 | 160 | 244 | 935 |
| **>1year** | **All** | 1217 | 433 | 691 | 2341 |
|  |  |  |  |  |  |
| **Specimen Collection** | **Cancer Type** | **TF<1%** | **1%≤TF<10%** | **TF≥10%** | **Total** |
| **Within 90days** | **NSCLC** | 551 | 295 | 246 | 1092 |
|  | **CRC** | 90 | 49 | 129 | 268 |
|  | **Breast** | 105 | 46 | 78 | 229 |
|  | **Pancreas** | 133 | 43 | 26 | 202 |
|  | **Prostate** | 53 | 15 | 49 | 117 |
|  | **Other** | 296 | 118 | 158 | 572 |
| **90days-1year** | **NSCLC** | 149 | 54 | 49 | 252 |
|  | **Breast** | 56 | 21 | 50 | 127 |
|  | **CRC** | 49 | 18 | 51 | 118 |
|  | **Pancreas** | 77 | 21 | 9 | 107 |
|  | **Prostate** | 32 | 8 | 25 | 65 |
|  | **Other** | 168 | 38 | 60 | 266 |
| **>1year** | **NSCLC** | 344 | 105 | 71 | 520 |
|  | **CRC** | 140 | 103 | 184 | 427 |
|  | **Breast** | 174 | 79 | 164 | 417 |
|  | **Prostate** | 164 | 38 | 146 | 348 |
|  | **Pancreas** | 111 | 13 | 15 | 139 |
|  | **Other** | 284 | 95 | 111 | 490 |

TF: tumor fraction, NSCLC: non-small cell lung cancer, CRC: colorectal cancer

**Supplementary Table 5: Baseline characteristics in MONSTAR-SCREEN cohort**

|  | **Total (N=459)**  **N (%)** |
| --- | --- |
| **Age** |  |
| < 65  ≥ 65 | 168 (37)  291 (63) |
| **Sex** |  |
| Male  Female | 307 (67)  152 (33) |
| **Cancer type** |  |
| Head and neck  Urothelial  Gastric  Renal cell  Melanoma  Esophageal  Colorectal  Endometrial  Other | 97 (21)  85 (19)  72 (16)  57 (12)  48 (10)  44 (9)  15 (3)  6 (2)  35 (8) |
| **Treatment line** |  |
| 1st  2nd  3^rd^- | 189 (41)  163 (36)  107 (23) |
| **MSI status** |  |
| High  MSS/MSI-Low  Unknown or NA | 30 (7)  124 (27)  305 (66) |
| **bTMB status** |  |
| ≥ 14 mut/Mb  < 14 mut/Mb | 49 (11)  410 (89) |
| **TMB status** |  |
| ≥ 10 mut/Mb  < 10 mut/Mb  Unknown or NA | 23 (5)  104 (23)  332 (72) |

MSI: microsatellite Instability, MSS: microsatellite Instability stable, NA: not applicable, bTMB: blood tumor mutational burden, TMB: tumor mutational burden

**Supplementary Table 6: Efficacy of immune checkpoint inhibitors according to tumor fraction**

|  | TF ≥10% | TF 1%–10% | TF <1% |
| --- | --- | --- | --- |
| ORR, % (95% CI) | 15.9 (10.7-23.0) | 26.3 (20.3-33.1) | 26.9 (20.0-35.1) |
| PFS, months (95% CI) | 2.1 (1.8-2.9) | 4.2 (3.5-5.7) | 5.8 (4.4-10.6) |

TF: tumor fraction, ORR: objective response rate, PFS: progression free survival

**Supplementary Table 7: Univariate and multivariate analysis of progression free survival by Cox proportional hazards model in patients with TF≥10%**

|  |  | **n** | **mPFS** | **Univariate analysis** | | | **Multivariate analysis** | |
| --- | --- | --- | --- | --- | --- | --- | --- | --- |
|  |  |  |  | **HR** | | **P-value** | **HR** | **P-Value** |
| Age | <65 | 46 | 2.7 | 1.0 (0.68-1.5) | | 0.93 |  |  |
|  | ≥65 | 92 | 2.1 | Reference |  | |  |  |
| Sex | Male | 96 | 2 | 0.99 (0.65-1.5) | | 0.95 |  |  |
|  | Female | 42 | 2.8 | Reference | |  |  |  |
| Cancer type | Hypermutated | 49 | 9.1 | 0.82 (0.55-1.2) | | 0.33 |  |  |
|  | Non-hypermutated | 89 | 3.8 | Reference | |  |  |  |
| Treatment line | 1^st^ | 31 | 3.4 | 0.61 (0.38-0.98) | | **0.04** | 0.59 (0.37-0.93) | **0.024** |
|  | 2^nd^- | 107 | 1.8 | Reference | |  | Reference |  |
| bTMB | ≥14 | 36 | 2.3 | 0.62 (0.39-0.98) | | **0.04** | 0.58 (0.36-0.93) | **0.025** |
|  | <14 | 102 | 2.1 | Reference | |  | Reference |  |

TF: tumor fraction, mPFS: median progression free survival, bTMB: blood tumor mutational burden

**Supplementary Table 8: Gene list of FoundationOneLiquid® CDx**

| ***ABL1***  ***[Exons 4-***  ***9]*** | *ACVR1B* | ***AKT1***  ***[Exon 3]*** | *AKT2* | *AKT3* | ***ALK***  ***[Exons 20-***  ***29, Introns***  ***18,19]*** | *ALOX12B* | *AMER1 (FAM123B)* | ***APC*** | ***AR*** |
| --- | --- | --- | --- | --- | --- | --- | --- | --- | --- |
| ***ARAF***  ***[Exons 4,***  ***5, 7, 11, 13,***  ***15, 16]*** | *ARFRP1* | *ARID1A* | *ASXL1* | ***ATM*** | ***ATR*** | *ATRX* | *AURKA* | *AURKB* | *AXIN1* |
| *AXL* | *BAP1* | *BARD1* | *BCL2* | *BCL2L1* | *BCL2L2* | *BCL6* | *BCOR* | *BCORL1* | *BCR**  *[Introns 8,*  *13, 14]* |
| ***BRAF***  ***[Exons 11-***  ***18****, Introns*  *7-10]* | ***BRCA1***  *[Introns 2,*  *7, 8, 12, 16,*  *19, 20]* | ***BRCA2***  *[Intron 2]* | *BRD4* | *BRIP1* | *BTG1* | *BTG2* | ***BTK***  ***[Exons 2,***  ***15]*** | *C11orf30 (EMSY)* | *C17orf39 (GID4)* |
| *CALR* | *CARD11* | *CASP8* | *CBFB* | *CBL* | ***CCND1*** | *CCND2* | *CCND3* | *CCNE1* | *CD22* |
| *CD70* | *CD74**  *[Introns 6-*  *8]* | *CD79A* | *CD79B* | ***CD274 (PD-L1)*** | *CDC73* | ***CDH1*** | ***CDK12*** | ***CDK4*** | ***CDK6*** |
| *CDK8* | *CDKN1A* | *CDKN1B* | ***CDKN2A*** | *CDKN2B* | *CDKN2C* | *CEBPA* | *CHEK1* | ***CHEK2*** | *CIC* |
| *CREBBP* | ***CRKL*** | *CSF1R* | *CSF3R* | *CTCF* | *CTNNA1* | ***CTNNB1***  ***[Exon 3]*** | *CUL3* | *CUL4A* | *CXCR4* |
| *CYP17A1* | *DAXX* | *DDR1* | ***DDR2***  ***[Exons 5,***  ***17, 18]*** | *DIS3* | *DNMT3A* | *DOT1L* | *EED* | ***EGFR***  ***[Introns 7,***  *15,* ***24-27]*** | *EP300* |
| *EPHA3* | *EPHB1* | *EPHB4* | ***ERBB2*** | ***ERBB3***  ***[Exons 3,***  ***6, 7, 8, 10,***  ***12, 20, 21,***  ***23, 24, 25]*** | *ERBB4* | *ERCC4* | *ERG* | ***ERRFI1*** | ***ESR1***  ***[Exons 4-***  ***8]*** |
| *ETV4**  *[Intron 8]* | *ETV5**  *[Introns 6,*  *7]* | ***ETV6****  ***[Introns 5,***  ***6]*** | *EWSR1**  *[Introns 7-*  *13]* | ***EZH2***  ***[Exons 4,***  ***16, 17, 18]*** | *EZR**  *[Introns 9-*  *11]* | *FAM46C* | *FANCA* | *FANCC* | *FANCG* |
| *FANCL* | *FAS* | *FBXW7* | *FGF10* | *FGF12* | *FGF14* | *FGF19* | *FGF23* | *FGF3* | *FGF4* |
| *FGF6* | ***FGFR1***  *[Introns 1,*  *5,* ***Intron***  ***17]*** | ***FGFR2***  *[Intron 1,*  ***Intron 17]*** | ***FGFR3***  ***[Exons 7, 9 (alternative designation exon 10),***  ***14, 18,***  ***Intron 17]*** | *FGFR4* | *FH* | *FLCN* | *FLT1* | ***FLT3***  ***[Exons 14,***  ***15, 20]*** | ***FOXL2*** |
| *FUBP1* | *GABRA6* | *GATA3* | *GATA4* | *GATA6* | ***GNA11***  ***[Exons 4,***  ***5]*** | *GNA13* | ***GNAQ***  ***[Exons 4,***  ***5]*** | ***GNAS***  ***[Exons 1, 8]*** | *GRM3* |
| *GSK3B* | *H3F3A* | *HDAC1* | *HGF* | *HNF1A* | ***HRAS***  ***[Exons 2,***  ***3]*** | *HSD3B1* | *ID3* | ***IDH1***  ***[Exon 4]*** | ***IDH2***  ***[Exon 4]*** |
| *IGF1R* | *IKBKE* | *IKZF1* | *INPP4B* | *IRF2* | *IRF4* | *IRS2* | *JAK1* | ***JAK2***  ***[Exon 14]*** | ***JAK3***  ***[Exons 5,***  ***11, 12, 13,***  ***15, 16]*** |
| *JUN* | *KDM5A* | *KDM5C* | *KDM6A* | *KDR* | *KEAP1* | *KEL* | ***KIT***  ***[Exons 8,9,11,12,***  ***13, 17****,*  *Intron 16]* | *KLHL6* | *KMT2A (MLL)*  *[Introns 6,*  *8-11,*  *Intron 7]* |
| *KMT2D (MLL2)* | ***KRAS*** | *LTK* | *LYN* | *MAF* | ***MAP2K1 (MEK1)***  ***[Exons 2,***  ***3]*** | ***MAP2K2 (MEK2)***  ***[Exons 2-***  ***4, 6, 7]*** | *MAP2K4* | *MAP3K1* | *MAP3K13* |
| *MAPK1* | *MCL1* | ***MDM2*** | *MDM4* | *MED12* | *MEF2B* | *MEN1* | *MERTK* | ***MET*** | *MITF* |
| *MKNK1* | *MLH1* | ***MPL***  ***[Exon 10]*** | *MRE11A* | *MSH2*  *[Intron 5]* | *MSH3* | *MSH6* | *MST1R* | *MTAP* | ***MTOR***  ***[Exons 19, 30, 39***  ***40, 43-45,***  ***47, 48, 53,***  ***56]*** |
| *MUTYH* | *MYB**  *[Intron 14]* | ***MYC***  ***[Intron 1]*** | *MYCL (MYCL1)* | ***MYCN*** | ***MYD88***  ***[Exon 4]*** | *NBN* | ***NF1*** | *NF2* | *NFE2L2* |
| *NFKBIA* | *NKX2-1 (TTF-1)* | *NOTCH1* | *NOTCH2*  *[Intron 26]* | *NOTCH3* | ***NPM1***  ***[Exons 4-***  ***6, 8, 10]*** | ***NRAS***  ***[Exons 2,***  ***3]*** | *NSD3 (WHSC1L1)* | *NT5C2* | ***NTRK1***  ***[Exons 14, 15,***  ***Introns 8-***  ***11]*** |
| *NTRK2*  *[Intron 12]* | ***NTRK3***  ***[Exons 16,***  ***17]*** | *NUTM1**  ***[Intron 1]*** | *P2RY8* | ***PALB2*** | *PARK2* | *PARP1* | *PARP2* | *PARP3* | *PAX5* |
| *PBRM1* | *PDCD1 (PD-1)* | ***PDCD1LG2 (PD-L2)*** | ***PDGFRA***  ***[Exons 12,***  ***18, Introns***  ***7, 9, 11]*** | ***PDGFRB***  ***[Exons 12-***  ***21, 23]*** | *PDK1* | *PIK3C2B* | *PIK3C2G* | ***PIK3CA***  ***[Exons 2, 3,***  ***5-8, 10, 14,***  ***19, 21***  ***(Coding Exons 1, 2,***  ***4-7, 9, 13,***  ***18, 20)]*** | *PIK3CB* |
| *PIK3R1* | *PIM1* | *PMS2* | *POLD1* | *POLE* | *PPARG* | *PPP2R1A* | *PPP2R2A* | *PRDM1* | *PRKAR1A* |
| *PRKCI* | *PTCH1* | ***PTEN*** | ***PTPN11*** | *PTPRO* | *QKI* | *RAC1* | *RAD21* | *RAD51* | *RAD51B* |
| *RAD51C* | *RAD51D* | *RAD52* | *RAD54L* | ***RAF1***  ***[Exons 3,***  ***4, 6, 7, 10,***  ***14, 15, 17,***  *Introns 4-8]* | *RARA*  *[Intron 2]* | ***RB1*** | *RBM10* | *REL* | ***RET***  ***[****Introns 7,*  *8,* ***Exons***  ***11, 13-16,***  ***Introns 9-***  ***11]*** |
| *RICTOR* | *RNF43* | ***ROS1***  ***[Exons 31,***  ***36-38, 40,***  ***Introns 31-***  ***35]*** | *RPTOR* | *RSPO2**  *[Intron 1]* | *SDC4**  *[Intron 2]* | *SDHA* | *SDHB* | *SDHC* | *SDHD* |
| *SETD2* | *SF3B1* | *SGK1* | *SLC34A2**  *[Intron 4]* | *SMAD2* | *SMAD4* | *SMARCA4* | *SMARCB1* | ***SMO*** | *SNCAIP* |
| *SOCS1* | *SOX2* | *SOX9* | *SPEN* | *SPOP* | *SRC* | *STAG2* | *STAT3* | ***STK11 (LKB1)*** | *SUFU* |
| *SYK* | *TBX3* | *TEK* | *TERC**  *{ncRNA}* | ***TERT****  ***{Promoter}*** | *TET2* | *TGFBR2* | *TIPARP* | *TMPRSS2**  *[Introns 1-3]* | *TNFAIP3* |
| *TNFRSF14* | ***TP53*** | *TSC1* | *TSC2* | *TYRO3* | *U2AF1* | ***VEGFA*** | *VHL* | *WHSC1* | *WTI* |
| *XPO1* | *XRCC2* | *ZNF217* | *ZNF703* |  | | | | | |

FoundationOne Liquid CDx interrogates 324 genes, including 309 genes with complete exonic (coding) coverage and 15 genes with only select non-coding coverage (indicated with an *); 75 genes (indicated in bold) are captured with increased sensitivity and have complete exonic (coding) coverage unless otherwise noted.
